# Cancer treatment fatigue and nutritional awareness in cancer patients: a latent profile analysis and predictors

**DOI:** 10.3389/fnut.2026.1707235

**Published:** 2026-01-26

**Authors:** Qinhong Meng, Yuxuan Du, Qiaoxiu Tan, Feifei He, Haojun Miao

**Affiliations:** 1 Department of Oncology, Haining Traditional Chinese Medicine Hospital, Haining, China; 2 Department of Oncology, Tongde Hospital of Zhejiang Province, Hangzhou, China

**Keywords:** cancer patients, cancer treatment fatigue, cancer-specific loneliness, death anxiety, nutritional awareness

## Abstract

**Purpose:**

Cancer requires long-term treatment, which imposes significant energy expenditure on the body, often exacerbating cancer-related fatigue among patients. Previous studies, adopting a variable-centered approach, have confirmed the relationship between cancer-related fatigue and nutrition management but have overlooked the relationship between cancer treatment fatigue and nutritional awareness, as well as their heterogeneity.

**Methods:**

From July to August 2025, we recruited 570 cancer patients using convenience sampling and employed latent profile analysis, single-variable analysis, and multivariable logistic regression to investigate the heterogeneity of cancer treatment fatigue and nutritional awareness, along with their influencing factors. Participants responded to measurement items related to variables such as cancer treatment fatigue, nutritional awareness, death anxiety, and cancer-specific loneliness, as well as provided necessary sociodemographic information.

**Results:**

Three profiles were identified: (1) High Cancer Treatment Fatigue–Low Nutritional Awareness (14.6%), characterized by severe fatigue and minimal attention to diet; (2) Moderate Cancer Treatment Fatigue–Moderate Nutritional Awareness (68.2%) as the largest and relatively balanced group; and (3) Low Cancer Treatment fatigue–High Nutritional awareness (17.2%), showing adaptive physical and behavioral traits. Younger age, lower education, smoking, and alcohol use were associated with membership in the high-fatigue/low-awareness group (*p* < 0.05).

**Conclusion:**

Distinct fatigue–nutrition coexistence patterns were demonstrated in oncology patients, suggesting that fatigue severity is closely intertwined with nutritional awareness. These findings provide evidence for incorporating personalized nutritional counseling and psychological fatigue-management programs into integrative cancer care to improve quality of life and treatment adherence.

## 1 Introduction

The rising incidence of cancer has underscored the urgency of comprehensive supportive care strategies (1). According to recent estimates by the World Health Organization, over 20 million new cancer cases were reported in 2022, with projections expecting this number to surge to 35 million by 2050 (2). The mortality of cancer patients is increasing due to population aging, smoking, alcohol consumption, obesity, and environmental exposure. This highlights the impact of screening, early detection, and multimodal therapies in consistently reducing mortality rates (3, 4). While medical advancements have transformed certain cancers from life-threatening acute illnesses into chronic conditions (5), thereby extending survival rates, the long-term side effects of these treatments continue to diminish patients’ quality of life. Among these, cancer treatment fatigue is a prevalent symptom, defined as distressing, persistent physical, emotional, or cognitive fatigue or tiredness associated with cancer or its treatment (6). This definition distinguishes cancer-related fatigue from ordinary fatigue through its severity, chronicity, and resistance to rest (7). Previous studies have shown that over half of cancer patients experience moderate to severe fatigue, particularly during active treatment phases such as chemotherapy or radiotherapy (8, 9). Cancer treatment fatigue often manifests as physical weakness, irritability, and attention deficits, severely impairing quality of life, work efficiency, and social roles (10). Despite its detrimental effects, cancer treatment fatigue is frequently underestimated and neglected, often due to the absence of objective quantifiable indicators, patient underreporting, and differences in social perception (11, 12). Therefore, addressing cancer treatment fatigue during cancer care is crucial for improving patients’ living standards. Recent research has linked cancer treatment fatigue to patients’ nutritional intake (13). Growing evidence indicates that systemic inflammation and nutritional imbalance jointly influence cancer prognosis. Recent inflammation- and nutrition-based prognostic indices, such as the Naples Prognostic Score, integrate serum albumin, cholesterol, and inflammatory ratios to predict survival outcomes. A study by Peker et al. (14) demonstrated the prognostic power of this composite score in non-small-cell lung cancer, underscoring the clinical relevance of coupling inflammation with nutritional status in oncology care. Furthermore, studies by Hou et al. (15) revealed that cachexia affects up to 80% of advanced cancer patients, further emphasizing the necessity of nutritional interventions in alleviating fatigue. Thus, nutritional awareness has emerged as a critical mediator, with rational dietary choices potentially mitigating fatigue by supporting metabolic recovery and reducing inflammation.

Nutritional awareness among cancer patients refers to the knowledge, attitudes, and behaviors related to diet and nutrition that are specifically adapted to the physiological and psychological needs of oncology care (16). Conceptually, it is a multidimensional construct integrating three interlinked domains: Cognitive knowledge, representing patients’ understanding of nutrient functions, dietary guidelines, and the relationship between diet and treatment outcomes (17); attitudinal disposition, reflecting motivation, perceived importance, and emotional commitment to maintaining adequate nutrition during cancer therapy; behavioral tendencies or intentions, capturing self-reported eating behaviors, food selection, and adherence to dietetic recommendations suitable for oncology patients (18). This multidimensional perspective recognizes that nutritional awareness is not merely informational but embodies the patient’s psychological engagement and practical application in everyday dietary choices (19). In this study, nutritional awareness is thus defined as an integrated psychosocial capacity that links cognitive understanding with motivational and behavioral execution in nutritional self-management during cancer treatment (20, 21). By optimizing energy reserves, immune function, and overall resilience, patients’ nutritional awareness plays a pivotal role in reducing treatment-related distress (22, 23). Malnutrition impairs cancer patients’ tolerance to treatment, immune capacity, and survival rates (24). Previous studies have confirmed that higher nutritional awareness is associated with better adherence to anticancer diets, reduced risk of cachexia, and improved quality of life (25, 26). For instance, a plant-based diet rich in antioxidants can alleviate oxidative stress induced by treatment, while adequate protein intake helps protect muscles from fatigue (26). However, fostering nutritional awareness among cancer patients faces numerous barriers, including misinformation about diet, cultural dietary norms, and treatment-induced anorexia, leading to prolonged malnutrition and exacerbating cancer-related fatigue.

While both cancer treatment fatigue and nutritional awareness hold significant importance, their heterogeneity among cancer patients has not been adequately explored. Traditional variable-centered analyses, such as regression models, have dominated research, revealing average associations while assuming population homogeneity and ignoring subgroups with distinct symptom profiles (27). Latent profile analysis (LPA), a person-centered approach, addresses this issue by categorizing individuals into homogeneous subgroups based on their response patterns to continuous indicators from cancer treatment fatigue and nutritional awareness scales, using fit indices such as BIC and entropy for model selection (28).

Nonetheless, the factors influencing cancer-related fatigue and nutritional awareness warrant attention, encompassing a range of sociodemographic, lifestyle, and psychosocial variables. A review of the literature suggests that these variables modulate symptom severity and awareness levels among cancer patients (29). Due to hormonal therapies and caregiving roles, women often report higher levels of cancer-related fatigue (30), while men may underreport symptoms but exhibit poorer nutritional awareness linked to traditional dietary habits (31). Older patients face exacerbated fatigue and reduced resilience due to comorbidities but may possess greater nutritional awareness gained from life experience (32). Married patients benefit from spousal encouragement, which can reduce fatigue and promote nutritional awareness, unlike single patients who endure heightened loneliness and suffering (33). Educational attainment positively correlates with nutritional awareness, as higher literacy levels facilitate better understanding of dietary guidelines (34). Cancer stage and diagnostic duration serve as clinical predictors (35); advanced-stage cancers (III-IV) amplify fatigue through tumor burden and intensified treatments (36), while longer post-diagnosis durations allow for adaptive nutritional awareness (37). Urban residents have greater access to resources that enhance nutritional awareness and reduce fatigue (37), whereas rural isolation exacerbates malnutrition and feelings of loneliness. From a lifestyle perspective, tobacco use impairs oxygen function and nutrient absorption, reducing the efficacy of nutritional awareness (38), while alcohol consumption disrupts sleep and metabolism, perpetuating fatigue cycles (39). Psychosocially, patients’ death anxiety can intensify the emotional component of fatigue and undermine motivation for self-nutritional care (40), while cancer-specific loneliness may promote disengagement and reduce social cues for healthy eating (41). Regrettably, these inferences are largely based on prior literature, with few studies validating the impact of factors such as gender, age, marital status, education, cancer stage, diagnostic duration, residence, income, smoking, alcohol consumption, death anxiety, and cancer-specific loneliness on the latent categories of fatigue and nutritional awareness.

Therefore, while prior studies have elucidated the average effects of cancer-related fatigue and nutritional awareness, few have employed latent profile analysis to identify subgroups or systematically examined the influence of factors such as gender, age, marital status, education, cancer stage, diagnostic duration, residence, income, smoking, alcohol consumption, death anxiety, and cancer-specific loneliness using multivariable logistic regression. This oversight overlooks potential interactions, such as how loneliness might amplify fatigue in low-nutritional awareness subgroups or how urban income might mitigate nutritional deficits. Addressing this gap is critical for advancing psycho-oncology; person-centered analyses can inform stratified interventions, enhancing quality of life and survival rates.

This study aims to: (1) identify latent profiles of cancer-related fatigue and nutritional awareness through latent profile analysis; (2) examine the distinct characteristics of these latent profiles; and (3) determine the factors influencing these latent categories using multivariable logistic regression. This research could inform personalized care plans for cancer patients, aligning with global calls for equitable cancer care. Ultimately, this study bridges methodological and substantive gaps, fostering evidence-based strategies to alleviate the dual burden of fatigue and nutritional deficiencies in the cancer trajectory.

## 2 Methods

### 2.1 Participants

This study adopted a cross-sectional observational design. Data collection was conducted in the Oncology Department of Tongde Hospital of Zhejiang Province and Haining Traditional Chinese Medicine Hospital, with ethical approval obtained from the hospital’s ethics committee (no.: 20250731).

#### 2.1.1 Data collection process

Researchers initially contacted the director of the Oncology Department at Tongde Hospital of Zhejiang Province and Haining Traditional Chinese Medicine Hospital, explaining the research procedure and potential benefits. Following consent, data collection was conducted in both the outpatient and inpatient sections of the Oncology Department. The data collection period was from July to August 2025. All measurement items were compiled into electronic questionnaires on the professional data collection platform, Credamo,^1^ with some paper questionnaires also retained. Participants were informed of the study protocol, and written informed consent was obtained before they completed the questionnaires.

#### 2.1.2 Sample size estimation

The minimum sample size was calculated using G*Power software (Version 3.1). Based on multivariate regression analysis, with an effect size of 0.10, a significance level of 0.05, and 13 predictor variables, a sample size of 277 was required to achieve a power of 0.95.

#### 2.1.3 Inclusion criteria

(1) Patients were diagnosed with cancer by the hospital’s pathology department. (2) Patients provided written informed consent. (3) No other severe comorbidities were present. (4) Patients had normal communication skills and no significant cognitive impairment. Cognitive impairment was assessed using the Mini-Mental State Examination, with a score below 23 considered indicative of impairment (42, 43).

#### 2.1.4 Exclusion criteria

(1) Patients exhibited significant cognitive impairment or unclear speech. (2) Questionnaires were incomplete or completed in less than 5 min. (3) Participants had recently participated in similar studies. (4) Informed consent was not provided.

Based on these criteria, convenience sampling was employed to recruit 600 participants. However, 12 participants were excluded due to unclear speech, 8 declined to provide informed consent, and 4 had mild cognitive impairment. After data cleaning, 576 questionnaires were retrieved. Four questionnaires with strong consistency and 2 incomplete questionnaires were further excluded, resulting in a final sample of 570 valid responses, with an effective response rate of 95%. Among these, 324 were male patients, and 246 were female patients. Detailed sociodemographic information is provided in Table 1.

**Table 1:** Summary of sociodemographic information.

Variables	Items	Numbers	Frequency
Gender	Male	324	56.80%
	Female	246	43.20%
Age	18 years old and under	59	10.40%
	19–35 years old	76	13.30%
	36–60 years old	222	38.90%
	Over 61 years of age	213	37.40%
Education background	Primary school and below	244	42.80%
	Middle school – High school	170	29.80%
	Bachelor degree or above	156	27.40%
Monthly income level	Under 3,000 ¥	136	23.90%
	3,001–5,000 ¥	205	36.00%
	5,001–8,000 ¥	184	32.30%
	More than 8,000 ¥	45	7.90%
Place of residence	Cities	266	46.70%
	Countryside	304	53.30%
Marriage	Married	452	79.30%
	Unmarried	60	10.50%
	Divorced	40	7.00%
	Widowed	18	3.20%
Staging of cancer	I	82	14.40%
	II	272	47.70%
	III	115	20.20%
	IV	101	17.70%
Smoking	Yes	352	61.80%
	No	218	38.20%
Drinking alcohol	Yes	274	48.10%
	No	296	51.90%

Prior to statistical analysis, the dataset was examined for item-level missing values. The overall proportion of missing data across all measurement items was 0.8%, which is below the recommended 5% threshold for potential bias (44). For isolated missing items (<5% per variable), mean substitution within each subscale was applied to preserve sample integrity and statistical power. Cases with missing values exceeding 20% within any single scale were listwise deleted to ensure data validity. Sensitivity analyses confirmed that imputing versus excluding these cases did not materially alter model estimates, supporting the robustness of the analytic results.

### 2.2 Research tools

#### 2.2.1 Cancer treatment fatigue scale

The measurement items for cancer treatment fatigue were adapted from the Cancer Fatigue Scale developed by Okuyama et al. (45), which consists of 15 items across three dimensions: physical, emotional, and cognitive states. The scale was translated into Chinese by Zhang et al. (46), who validated its cultural adaptability and psychometric properties. The scale has been widely applied to various cancer populations (47, 48), confirming its operational simplicity and suitability for diverse cancer groups. A 5-point Likert scale was used for scoring, with higher scores indicating greater fatigue (1 = “Strongly Disagree,” 5 = “Strongly Agree”). Confirmatory factor analysis (CFA) using AMOS 29.0 yielded good model fit (*χ*^2^/df = 2.271, GFI = 0.955, AGFI = 0.939, CFI = 0.970, TLI = 0.965, RMSEA = 0.047). Reliability analysis revealed a Cronbach’s alpha of 0.925, indicating excellent reliability.

#### 2.2.2 Nutritional awareness scale

The nutritional awareness items were adapted from the scale developed by van Dillen et al. (49), which contains 17 items. A back-translation procedure was used to ensure cultural adaptability: one bilingual scholar translated the English version into Chinese, and another translated it back into English without reference to the original (50). A 5-point Likert scale was used for scoring, with higher scores indicating greater nutritional awareness (1 = “Strongly Disagree,” 5 = “Strongly Agree”). CFA using AMOS 29.0 confirmed good model fit (*χ*^2^/df = 2.443, GFI = 0.936, AGFI = 0.918, CFI = 0.948, TLI = 0.941, RMSEA = 0.050). Reliability analysis yielded a Cronbach’s alpha of 0.910, indicating strong reliability.

In this study, nutritional awareness was treated as a latent continuous indicator integrating cognitive, attitudinal, and behavioral dimensions. Mean item scores across all 17 items were used as manifest variables in the latent profile analysis (LPA), enabling identification of patient subgroups characterized by different coexisting patterns of nutritional awareness and cancer treatment fatigue.

#### 2.2.3 Death anxiety scale

The death anxiety items were adapted from the scale developed by Templer (51), which includes 15 items. The scale was translated into Chinese by Yang et al. (52) and validated for cultural adaptability and reliability in Chinese cancer patients (53). It has been widely used in Chinese cancer populations, including breast cancer patients (54). A 5-point Likert scale was used for scoring, with higher scores indicating greater death anxiety (1 = “Strongly Disagree,” 5 = “Strongly Agree”). CFA using AMOS 29.0 showed good model fit (*χ*^2^/df = 2.699, GFI = 0.944, AGFI = 0.926, CFI = 0.942, TLI = 0.932, RMSEA = 0.055). Reliability analysis revealed a Cronbach’s alpha of 0.892, indicating good reliability.

#### 2.2.4 Cancer-specific loneliness

The cancer-specific loneliness items were adapted from the scale developed by Adams et al. (55), which contains 15 items. The scale was applied to Chinese nasopharyngeal cancer patients by Luo et al. (56) and translated into Chinese by Cui (57). A 5-point Likert scale was used for scoring, with higher scores indicating greater cancer-specific loneliness (1 = “Strongly Disagree,” 5 = “Strongly Agree”). CFA using AMOS 29.0 yielded good model fit (*χ*^2^/df = 3.805, GFI = 0.919, AGFI = 0.892, CFI = 0.893, TLI = 0.875, RMSEA = 0.070). Reliability analysis revealed a Cronbach’s alpha of 0.874, indicating good reliability.

### 2.3 Data analysis

First, we used AMOS 29.0 software to analyze the model fit for each variable. Subsequently, we used SPSS 27.0 software for reliability testing, common method bias testing, descriptive analysis, and correlation analysis. Next, we used Mplus 8.3 software to conduct latent profile analysis to identify potential categories of nutritional awareness and cancer treatment fatigue among cancer patients. We utilized the scores from the 17 nutritional awareness items and the 15 cancer treatment fatigue items as manifest variables. We then constructed models with 1 to 5 latent categories in Mplus 8.3. The final model was determined based on fit indices such as AIC, BIC, aBIC, Entropy, and *p*-values (LMR and BLRT). For the single-variable analysis, we examined factors such as gender, age, marital status, education, cancer stage, and diagnostic duration to determine the proportions of latent categories for nutritional awareness and cancer treatment fatigue. We then used variance analysis to test the differences in death anxiety and cancer-specific loneliness across the latent categories of nutritional awareness and cancer treatment fatigue. We used *F*-values, *χ*^2^ and *p*-values to identify the global effect size. Finally, a multinomial logistic regression analysis was conducted to identify demographic, clinical, and behavioral factors associated with latent profile membership. The three-profile categorical variable derived from the LPA was treated as a nominal outcome, with the moderate fatigue–moderate nutritional awareness group serving as the reference category. Independent variables included age, sex, education, cancer type, smoking, alcohol consumption, and other relevant covariates. Adjusted odds ratios (ORs) and 95% confidence intervals (CIs) were reported. This approach appropriately accounts for the non-ordinal, nominal nature of the latent profile variable.

The model fit indices used were: *χ*^2^/*df* to assess overall model fit; GFI to indicate the proportion of variance and covariance explained by the model; AGFI as an adjustment to GFI considering model degrees of freedom; TLI to compare the target model with a baseline model; RMSEA as an absolute fit index, considering the difference between the theoretical model and a perfectly fitting model; CFI to assess the improvement in fit of the target model over the baseline model. For latent profile analysis, the indices used were AIC, BIC, aBIC, Entropy, LMR, and BLRT. Lower values of AIC, BIC, and aBIC indicate better model fit. Entropy values greater than 0.8 are generally considered to indicate good classification quality. The Lo–Mendell–Rubin Adjusted Likelihood Ratio Test (LMR) is a nested model comparison test based on likelihood ratio; *p* < 0.05 indicates significance. The Bootstrapped Likelihood Ratio Test (BLRT) uses a parametric Bootstrap method to simulate the distribution of log-likelihood differences under a k-1 model, providing a more accurate *p*-value; *p* < 0.05 indicates significance.

All variables were screened for missing values prior to statistical analysis. Missingness patterns were assessed using Little’s MCAR test (*χ*^2^ = 47.62, *p* = 0.214), indicating that the data were missing completely at random (MCAR). For continuous variables (e.g., Cancer Treatment Fatigue and Nutritional Awareness scores), mean imputation based on subscale means was adopted for ≤5% missing items per participant. For categorical demographic variables, mode substitution was applied if only one or two responses were absent. Participants with extensive missing data (>20% of any scale) were excluded listwise. This hybrid approach ensured completeness while minimizing potential estimation bias in latent profile analysis and logistic regression. All subsequent analyses were based on the final dataset of 570 participants.

## 3 Results

### 3.1 Common method bias analysis

Common method bias often arises from single-source bias, measurement biases under the same context, or the similarity of measurement methods, leading to artificial covariance between predictor and criterion variables. To mitigate this, we informed participants that their data would be strictly confidential and anonymous during collection to reduce social desirability bias and, consequently, common method bias. Additionally, we retained reverse-coded items in the questionnaire design to further reduce common method bias.

We then subjected all measurement items to exploratory factor analysis, conducting a Harman single-factor test. The results showed that the first factor explained 24.302% of the variance, which was below the critical threshold of 40%. Therefore, no significant common method bias was detected in this study.

### 3.2 Descriptive statistics and correlation analysis

We conducted descriptive statistics and correlation analysis for all sociodemographic information and study variables, as shown in Table 2. The results indicated: The mean score for cancer treatment fatigue was 3.014 (SD = 0.798), indicating moderate fatigue among participants. The mean score for nutritional awareness was 3.194 (SD = 0.709), indicating moderate nutritional awareness. The mean scores for death anxiety and cancer-specific loneliness were 3.203 (SD = 0.694) and 3.270 (SD = 0.661), respectively, both indicating moderate levels.

**Table 2:** Results of correlation analysis and descriptive statistics for all variables.

Variables	M	SD	1	2	3	4	5	6	7	8	9	10	11	12	13
1. Gender	–	–	1												
2. Age	–	–	−0.071	1											
3. Education background	–	–	−0.106*	0.062	1										
4. Monthly income level	–	–	0.101*	−0.008	−0.132**	1									
5. Place of residence	–	–	−0.010	0.011	−0.030	0.056	1								
6. Marriage	–	–	−0.020	0.031	−0.049	−0.044	−0.026	1							
7. Cancer staging	–	–	0.024	0.047	0.084*	0.024	−0.012	−0.073	1						
8. Smoking	–	–	0.063	0.150**	0.099*	−0.196**	−0.009	0.009	0.024	1					
9. Drinking alcohol	–	–	0.423**	−0.054	−0.048	−0.021	0.036	0.044	0.030	0.143**	1				
10. Cancer treatment fatigue	3.014	0.798	−0.270**	0.073	0.006	0.125**	−0.081	−0.001	0.028	0.233**	−0.229**	1			
11. Nutritional awareness	3.194	0.709	0.214**	0.036	0.053	−0.044	0.016	0.005	−0.005	0.081	0.084*	−0.518**	1		
12. Death anxiety	3.203	0.694	−0.185**	0.087*	−0.005	0.129**	−0.046	−0.088*	0.065	0.109**	−0.107*	0.569**	−0.270**	1	
13. Cancer-specific loneliness	3.270	0.661	−0.144**	0.007	−0.013	0.093*	−0.095*	−0.097*	0.080	0.072	−0.147**	0.416**	−0.187**	0.648**	1

***p < 0.001; **p < 0.01; *p < 0.05.

Cancer treatment fatigue was strongly negatively correlated with nutritional awareness (*r* = −0.518, *p* < 0.001, 95% CI = [−0.576, −0.456]), indicating that higher treatment fatigue was associated with lower nutritional awareness. Cancer treatment fatigue was also significantly positively correlated with death anxiety (*r* = 0.569, *p* < 0.001, 95% CI = [0.511, 0.622]) and cancer-specific loneliness (*r* = 0.416, *p* < 0.001, 95% CI = [0.345, 0.481]). Nutritional awareness was significantly negatively correlated with death anxiety (*r* = −0.270, *p* < 0.001, 95% CI = [−0.345, −0.192]) and cancer-specific loneliness (*r* = −0.187, *p* < 0.001, 95% CI = [−0.265, −0.107]). Sociodemographic information and lifestyle factors also showed significant correlations. Smoking was positively correlated with cancer treatment fatigue (*r* = 0.233, *p* < 0.001) and death anxiety (*r* = 0.109, *p* < 0.001); while gender was negatively correlated with cancer treatment fatigue (*r* = −0.270, *p* < 0.001).

### 3.3 Latent profile analysis

This study used Mplus software with robust maximum likelihood estimation to address potential non-normality of the data and provided standard errors with bias correction. Specifically, we used latent profile analysis to evaluate models with 1 to 5 latent profiles based on the measurement items for cancer treatment fatigue and nutritional awareness, aiming to identify the model with the best fit. The fit indices for the models are summarized in Table 3. Models were estimated using 500 random starts and 100 final-stage optimizations to ensure convergence on global maxima. We observed that AIC, BIC, and aBIC values decreased as the number of latent categories increased, indicating improving model fit. However, the entropy values for 4 latent categories (0.961–0.990) were very high, indicating clear classification. Additionally, both the LMR and BLRT values remained statistically significant (*p* < 0.001) for models with 2 to 4 latent profiles.

**Table 3:** Fit indices for latent profile analysis of cancer treatment fatigue and nutritional awareness.

Profile	AIC	BIC	aBIC	Entropy	LMR (P)	BLRT (P)	Smallest proportion per class
1	56069.735	56347.856	56144.685				
2	52834.386	53255.913	52947.981	0.985	<0.001	<0.001	0.819/0.181
3	50320.249	50885.182	50472.490	0.990	<0.001	<0.001	0.146/0.172/0.682
4	49138.645	49846.984	49329.532	0.961	<0.001	<0.001	0.463/0.144/0.160/0.233
5	48818.028	49669.773	49047.561	0.967	0.0006	<0.001	0.160/0.016/0.452/0.144/0.228

Upon further comparison, we found that the three-profile model was the optimal choice (AIC = 50320.249, BIC = 50885.182, aBIC = 50472.490, LMR(P) < 0.001, BLRT(P) < 0.001). The entropy value for the three-profile model (Entropy = 0.990) was significantly higher than those for the other models, demonstrating clearer classification. Therefore, selecting three latent profiles was more reasonable and substantively meaningful.

### 3.4 Subgroup classification

**Figure 1:**
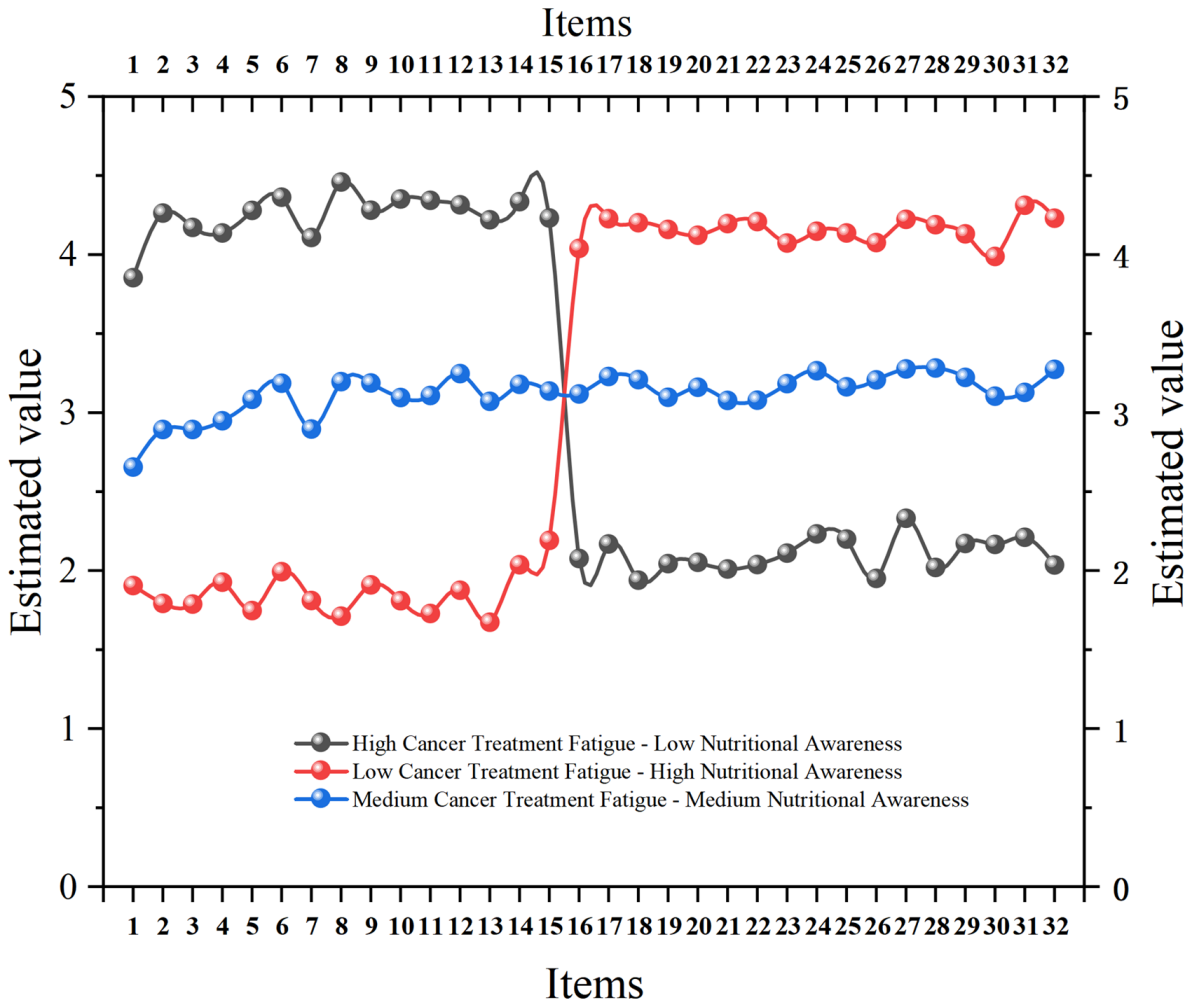
Latent profiles of cancer treatment fatigue and nutritional awareness among oncology patients. The x-axis presents standardized item scores across the two measurement domains: Items 1–15 correspond to cancer treatment fatigue, encompassing physical (items 1–3, 6, 9, 12, 15), emotional (items 5, 8, 11, 14), and cognitive (items 4, 7, 10, 13) dimensions. Items 16–32 reflect nutritional awareness. The y-axis indicates the standardized mean response for each item. The three colored curves represent the identified latent subgroups derived from the latent profile analysis: Profile 1: High cancer treatment fatigue – low nutritional awareness (14.6%); Profile 2: Low cancer treatment fatigue – high nutritional awareness (17.2%); Profile 3: Moderate cancer treatment fatigue – moderate nutritional awareness (68.2%). Higher values on the y-axis indicate greater symptom intensity (for fatigue items) or stronger awareness and engagement (for nutritional items).

Based on the latent profile analysis results in section 3.3, we identified three latent categories for cancer treatment fatigue and nutritional awareness. We conducted a visual analysis of the three latent categories, as shown in Figure 1. The first subgroup was named “High Cancer Treatment Fatigue—Low Nutritional Awareness,” accounting for approximately 14.6% of the sample. The second subgroup was labeled “Moderate Cancer Treatment Fatigue—Moderate Nutritional Awareness,” representing 68.2%. The third subgroup was termed “Low Cancer Treatment Fatigue—High Nutritional Awareness,” comprising 17.2%.

### 3.5 Single-factor analysis of cancer treatment fatigue and nutritional awareness

To further analyze differences in the latent categories of cancer treatment fatigue and nutritional awareness across sociodemographic factors, death anxiety, and cancer-specific loneliness, we conducted single-factor analysis, as shown in Table 4. The results indicated significant differences in cancer treatment fatigue and nutritional awareness across the following variables: Gender (*χ*^2^ = 53.974, *p* < 0.001), age (*χ*^2^ = 43.311, *p* < 0.001), monthly income level (*χ*^2^ = 14.98, *p* < 0.02) and Cancer stage (*χ*^2^ = 24.193, *p* < 0.001). Single-factor variance analysis revealed significant differences in death anxiety (*F* = 138.597, *p* < 0.001) and cancer-specific loneliness (*F* = 79.578, *p* < 0.001). However, no significant differences were found for education level (*χ*^2^ = 2.833, *p* = 0.586), residence (*χ*^2^ = 5.01, *p* = 0.082), or marital status (*χ*^2^ = 5.535, *p* = 0.447).

**Table 4:** Single-factor analysis of cancer treatment fatigue and nutritional awareness latent categories.

Variables	Items	Class 1(14.6%)	Class 2 (17.2%)	Class 3 (68.2%)	*χ*^2^/*F*	*p*
Gender	Male	60	18	180	53.974	<0.001
	Female	23	81	208		
Age	18 years old and under	1	20	38	43.311	<0.001
	19–35 years old	24	12	40		
	36–60 years old	39	29	154		
	Over 61 years of age	19	38	156		
Education background	Primary school and below	36	40	168	2.833	0.586
	Middle school – High school	25	36	109		
	Bachelor degree or above	22	23	111		
Monthly income level	Under 3,000 ¥	16	31	84	14.98	0.02
	3,001–5,000 ¥	21	32	152		
	5,001–8,000 ¥	33	29	122		
	More than 8,000 ¥	13	7	30		
Place of residence	Cities	48	46	172	5.01	0.082
	Countryside	35	53	216		
Marriage	Married	65	77	310	5.535	0.447
	Unmarried	7	9	44		
	Divorced	9	10	21		
	Widowed	2	3	13		
Staging of cancer	I	13	14	55	24.193	<0.001
	II	26	38	208		
	III	19	23	73		
	IV	25	24	52		
Smoking	Yes	67	82	203	45.883	<0.001
	No	16	17	185		
Drinking alcohol	Yes	65	51	158	39.276	<0.001
	No	18	48	230		
Death anxiety		3.949 ± 0.823	2.538 ± 0.735	3.213 ± 0.442	138.597	<0.001
Cancer treatment loneliness		3.969 ± 0.714	2.914 ± 0.848	3.211 ± 0.459	79.578	<0.001

### 3.6 Multinomial logistic regression analysis of factors influencing cancer treatment fatigue and nutritional awareness

We used the “Moderate Cancer Treatment Fatigue—Moderate Nutritional Awareness” group (Class 3) as the reference category and conducted multinomial logistic regression analysis, incorporating variables that showed statistical significance in the single-factor analysis as predictors. The results are presented in Table 5. Prior to the analysis, we assessed multicollinearity; all variance inflation factor (VIF) values ranged between 1.012 and 1.810, well below the threshold of 5, indicating no substantial multicollinearity.

**Table 5:** Logistic regression analysis of factors influencing latent profiles of cancer treatment fatigue and nutritional awareness.

Classification	Variables	Items	Regression coefficient	Standard error	Wald *χ*^2^	*p*	OR	LLCI	ULCI
High cancer treatment fatigue – low nutritional awareness (14.6%)	Death anxiety		0.871	0.354	6.05	0.014	2.39	1.194	4.786
	Cancer treatment loneliness		1.466	0.392	13.986	<0.001	4.331	2.009	9.338
	Gender	Male	1.17	0.412	8.054	0.005	3.221	1.436	7.223
		Female (refer)							
	Age	18 years old and under	−0.315	1.122	0.079	0.779	0.73	0.081	6.58
		19–35 years old	1.37	0.485	7.968	0.005	3.936	1.52	10.19
		36–60 years old	0.563	0.397	2.015	0.156	1.756	0.807	3.823
		Over 61 years of age (refer)							
	Monthly income level	Under 3,000 ¥	−0.813	0.626	1.685	0.194	0.443	0.13	1.514
		3,001–5,000 ¥	−1.104	0.583	3.585	0.058	0.332	0.106	1.039
		5,001–8,000 ¥	−0.382	0.536	0.509	0.475	0.682	0.239	1.95
		More than 8,000 ¥ (refer)							
	Staging of cancer	I	−0.634	0.577	1.205	0.272	0.531	0.171	1.645
		II	−1.355	0.445	9.263	0.002	0.258	0.108	0.617
		III	−0.41	0.495	0.687	0.407	0.664	0.252	1.75
		IV (refer)							
	Smoking	Yes	1.322	0.382	11.981	0.001	3.749	1.774	7.924
		No (refer)							
	Alcohol consumption	Yes	0.765	0.379	4.072	0.044	2.148	1.022	4.514
		No (refer)							
Low cancer treatment fatigue – high nutritional awareness (17.2%)	Death anxiety		−1.766	0.284	38.591	<0.001	0.171	0.098	0.299
	Cancer treatment loneliness		0.035	0.257	0.018	0.893	1.035	0.626	1.712
	Gender	Male	−2.369	0.395	36.01	<0.001	0.094	0.043	0.203
		Female (refer)							
	Age	18 years old and under	0.26	0.418	0.387	0.534	1.297	0.572	2.941
		19–35 years old	−0.137	0.489	0.079	0.779	0.872	0.334	2.272
		36–60 years old	0.024	0.34	0.005	0.943	1.025	0.526	1.995
		Over 61 years of age (refer)							
	Monthly income level	Under 3,000 ¥	0.748	0.594	1.586	0.208	2.114	0.659	6.775
		3,001–5,000 ¥	0.191	0.582	0.108	0.742	1.211	0.387	3.79
		5,001–8,000 ¥	−0.286	0.563	0.259	0.611	0.751	0.249	2.262
		More than 8,000 ¥ (refer)							
	Staging of cancer	I	−1.117	0.508	4.83	0.028	0.327	0.121	0.886
		II	−0.936	0.397	5.55	0.018	0.392	0.18	0.854
		III	−0.667	0.456	2.145	0.143	0.513	0.21	1.253
		IV (refer)							
	Smoking	Yes	1.787	0.394	20.56	<0.001	5.969	2.758	12.921
		No (refer)							
	Alcohol consumption	Yes	1.506	0.34	19.601	<0.001	4.508	2.315	8.781
		No (refer)							

Comparison of the “High Cancer Treatment Fatigue—Low Nutritional Awareness” Group (Class 1) with the Reference Group (Class 3). Men were more likely than women to belong to Class 1 (OR = 3.221, 95% CI = [1.436, 7.223], *p* = 0.005). Compared to participants aged 61 and older, those aged 19–35 were more likely to belong to Class 1 (OR = 3.936, 95% CI = [1.52, 10.19], *p* = 0.005). Smoking (OR = 3.749, 95% CI = [1.774, 7.924], *p* = 0.001) and alcohol consumption (OR = 2.148, 95% CI = [1.022, 4.514], *p* = 0.044) significantly increased the likelihood of belonging to Class 1. Higher death anxiety (OR = 2.39, 95% CI = [1.194, 4.786], *p* = 0.014) and cancer-specific loneliness (OR = 4.331, 95% CI = [2.009, 9.338], *p* < 0.001) were also strong predictors. In contrast, compared to patients with Stage IV cancer, those diagnosed at Stage II were significantly less likely to belong to Class 1 (OR = 0.258, 95% CI = [0.108, 0.617], *p* = 0.002).

**Figure 2:**
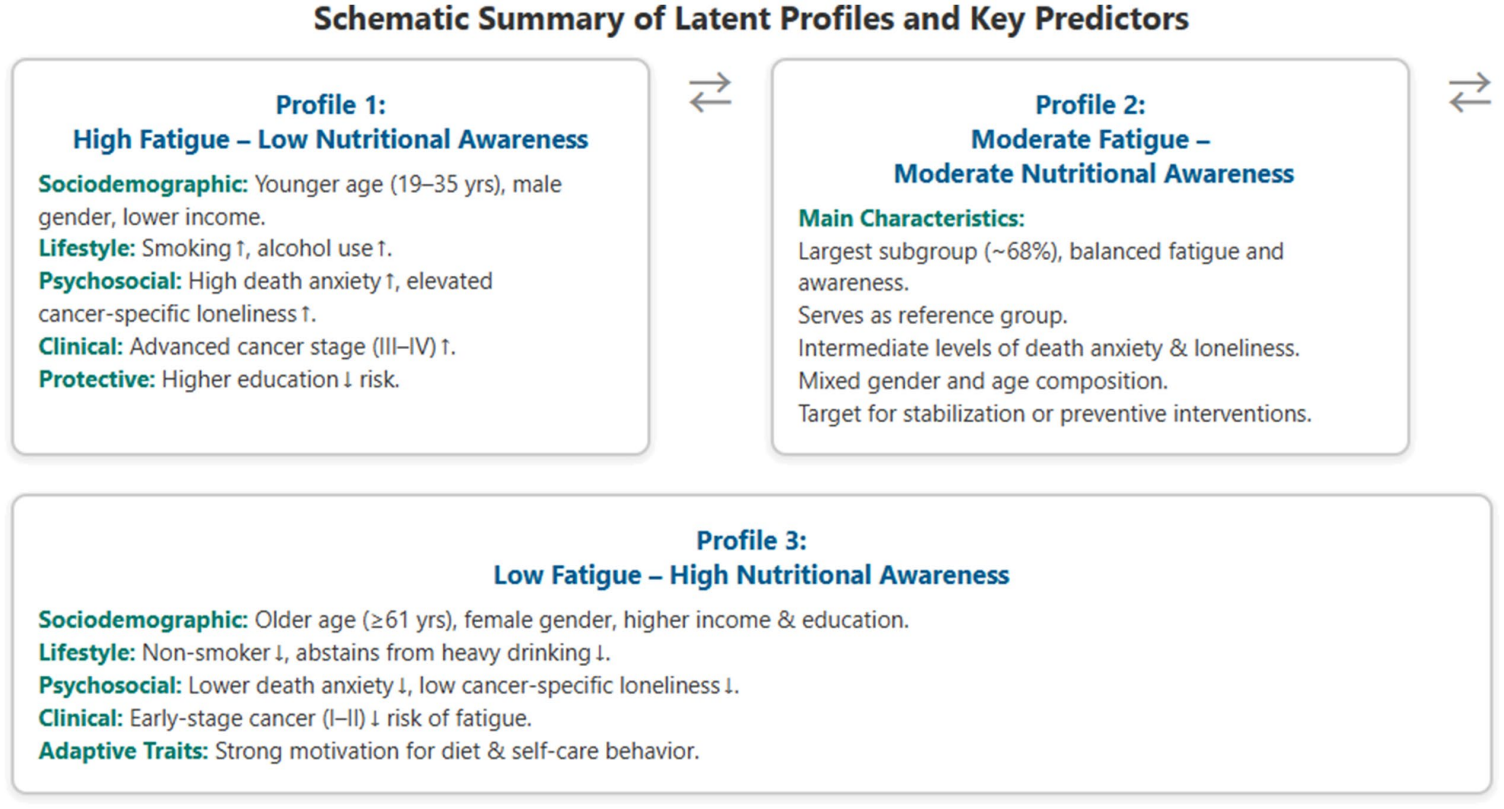
Schematic summary of latent profiles and key predictors. Arrows depict the continuum of fatigue–awareness severity across profiles. Upward arrows (↑) indicate variables increasing profile membership likelihood; downward arrows (↓) indicate protective associations as identified in logistic regression (*p* < 0.05). Diagram synthesized from multinomial regression results of the present study.

Comparison of the “Low Cancer Treatment Fatigue—High Nutritional Awareness” Group (Class 2) with the Reference Group (Class 3). Women were significantly more likely than men to belong to Class 2 (OR = 0.094, 95% CI = [0.043, 0.203], *p* < 0.001). Smoking (OR = 5.969, 95% CI = [2.758, 12.921], *p* < 0.001) and alcohol consumption (OR = 4.508, 95% CI = [2.315, 8.781], *p* < 0.001) significantly increased the likelihood of belonging to Class 2. Higher death anxiety was associated with a reduced likelihood of belonging to this group (OR = 0.171, 95% CI = [0.098, 0.299], *p* < 0.001). Early cancer stage was protective: compared to Stage IV patients, those diagnosed at Stage I (OR = 0.327, 95% CI = [0.121, 0.886], *p* = 0.028) and Stage II (OR = 0.392, 95% CI = [0.18, 0.854], *p* = 0.018) were significantly less likely to belong to Class 2 (Figure 2).

## 4 Discussion

### 4.1 Latent profile analysis and underlying mechanisms

Latent profile analysis revealed three distinct subgroups characterized by concurrent patterns of cancer treatment fatigue and nutritional awareness: High Cancer Treatment Fatigue–Low Nutritional Awareness (14.6%), Moderate Cancer Treatment Fatigue–Moderate Nutritional Awareness (68.2%), and Low Cancer Treatment Fatigue–High Nutritional Awareness (17.2%). Model fit indices confirmed the robustness and clarity of classification. The predominance of the moderate profile indicates that most patients maintain a relatively balanced but not optimal state, whereas the high- and low-extreme profiles represent distinct vulnerability and resilience patterns. This heterogeneity underscores the advantages of person-centered methods over traditional variable-centered analyses, which often average out individual differences.

Mechanistically, the bidirectional interplay between fatigue and nutritional awareness provides an explanatory framework. In the high-fatigue/low-awareness group, insufficient nutritional knowledge and maladaptive eating behaviors exacerbate metabolic imbalance, inflammation, and cachexia, which further intensify fatigue—a self-reinforcing cycle of physical and motivational decline. In contrast, individuals in the low-fatigue/high-awareness group demonstrate adaptive regulation, active dietary management, and higher health engagement that buffer against treatment-related exhaustion. The moderate group may represent a transitional state prone to either deterioration or adaptation depending on psychosocial and behavioral moderators.

Overall, these latent profiles reveal how nutritional awareness acts not only as a correlate of cancer-related fatigue but also as a modifiable mediator that influences recovery, treatment adherence, and overall well-being. Recognizing these subgroups provides a data-driven foundation for personalized management strategies aimed at simultaneously reducing fatigue and strengthening self-care awareness among oncology patients.

### 4.2 Factors influencing potential subgroups

#### 4.2.1 Death anxiety

Death anxiety significantly influenced the differences between latent profiles, with higher levels increasing the likelihood of belonging to the high cancer treatment fatigue–low nutritional awareness group (OR = 2.39, *p* = 0.014) while reducing membership in the low cancer treatment fatigue–high nutritional awareness group (OR = 0.171, *p* < 0.001). This finding aligns with terror management theory, which posits that existential fear amplifies emotional and physical distress, potentially manifesting as heightened fatigue and diminished motivation for health-promoting behaviors. In oncology, death anxiety is typically positively correlated with cancer treatment fatigue and negatively associated with quality of life indicators, including nutritional awareness. The moderate level of death anxiety observed in this study (M = 3.203, SD = 0.844) suggests a widespread impact, potentially exacerbated by cultural factors such as filial piety and stoicism in Chinese patients, leading to avoidance of health-promoting activities. Mechanistically, elevated death anxiety may disrupt autonomic nervous system regulation, increase cortisol levels, exacerbate inflammatory responses and fatigue, and erode the cognitive resources necessary for nutritional awareness, such as understanding dietary guidelines. Therefore, comprehensive interventions targeting death anxiety in cancer patients are essential to optimize their latent profile transitions. Meaning-centered psychotherapy, which has been shown to reduce death anxiety and improve fatigue outcomes in advanced cancer trials, may enhance nutritional awareness through goal-oriented coping, potentially transitioning patients from high-risk to moderate or protective profiles. Routine screening for death anxiety using validated instruments in oncology outpatient settings could enable early identification of high-risk subgroups and facilitate personalized care plans integrating anxiety management with nutrition counseling.

#### 4.2.2 Cancer treatment loneliness

Cancer treatment loneliness emerged as a strong predictor, significantly increasing the likelihood of belonging to the high cancer treatment fatigue–low nutritional awareness group (OR = 4.331, *p* < 0.001), while showing no significant association with the low cancer treatment fatigue–high nutritional awareness group (OR = 1.035, *p* = 0.893). This differential effect supports social isolation theory, where perceived disconnection amplifies symptom severity and impairs health behaviors. In cancer populations, loneliness exacerbates fatigue and diminishes nutritional awareness, as social cues for healthy eating are reduced during social withdrawal. These findings advocate for interventions targeting loneliness to promote favorable profile transitions, such as fostering social integration and peer support networks that share nutrition strategies, reducing fatigue in vulnerable subgroups.

#### 4.2.3 Age

Contrary to the common assumption that older patients are more vulnerable, this study found a significantly higher proportion of younger patients (aged 19–35) in the high fatigue–low nutritional awareness group (OR = 3.936, *p* = 0.005). Younger patients may face psychological maladjustment due to the dual pressures of career and family responsibilities, leading to greater fatigue and poorer self-management behaviors. Additionally, younger patients may lack knowledge about the long-term implications of cancer and its treatments, resulting in lower nutritional awareness, whereas older patients may exhibit better nutritional behaviors due to greater life experience and chronic disease management knowledge. However, older patients also face unique challenges, such as comorbidities and declining physical function, which are more common in the moderate fatigue–moderate awareness group. Although aging may accompany cognitive and physical decline, life experience and social support may partially compensate for these deficits. Thus, the impact of age on cancer treatment fatigue and nutritional awareness must be understood through a life-course perspective that considers social role changes. We recommend enhanced psychological support and health education for younger patients to improve their understanding of treatment side effects and the importance of nutrition; for older patients, addressing comorbidities and functional maintenance through family and community support is crucial to enhance the feasibility and sustainability of nutritional behaviors.

#### 4.2.4 Gender

Gender was a significant factor distinguishing latent profiles, with male patients more likely to belong to the high fatigue–low nutritional awareness group (OR = 3.221, *p* = 0.005) and female patients more likely to belong to the low fatigue–high nutritional awareness group (OR = 0.094, *p* < 0.001). Men generally exhibit lower willingness to report fatigue and seek help, potentially underestimating symptom severity and engaging in poorer fatigue and nutritional behaviors. In contrast, women tend to be more attentive to health information and more willing to engage in self-management behaviors, demonstrating higher nutritional awareness and better fatigue regulation. Gender differences also reflect communication and support disparities within healthcare systems. For instance, female patients often maintain more active communication with healthcare providers, making them more likely to receive nutrition advice and psychological support. Therefore, targeted health communication strategies are needed for male cancer patients, such as digital health tools or peer support programs, to enhance their access to health information and self-management motivation.

#### 4.2.5 Monthly income level

Monthly income level, as a key indicator of socioeconomic status, showed significant effects on nutritional awareness and treatment fatigue profiles. Although income itself had limited independent effects in the multinomial logistic regression, its influence was mediated by factors such as resource availability and health inequality. Low-income patients (monthly income <3,000 CNY) were more likely to experience food insecurity, lack of nutrition knowledge, and limited access to medical resources, which may collectively contribute to their membership in the high fatigue–low nutritional awareness group. Conversely, higher income could provide greater access to healthy foods, nutrition counseling, and supportive services, fostering the formation of the low fatigue–high nutritional awareness profile. Notably, income effects often interact with other factors; for example, low-income rural patients may face compounded challenges such as geographic isolation and information scarcity, further exacerbating health disparities. Thus, addressing health inequalities requires multifaceted interventions, including enhanced community nutrition education, improved access to primary healthcare, and policy support to reduce health-related social determinants.

#### 4.2.6 Cancer stage

Cancer stage was a critical clinical predictor of treatment fatigue and nutritional awareness. This study revealed that, compared to Stage IV patients, Stage II patients were significantly less likely to belong to the high fatigue–low nutritional awareness group (OR = 0.258, *p* = 0.002), while patients diagnosed at Stage I or II were also less likely to belong to the low fatigue–high nutritional awareness group. This suggests that advancing disease severity and intensified treatment burden exacerbate fatigue and may reduce nutritional awareness due to metabolic disorders and decreased appetite. Advanced cancer patients often experience cachexia and systemic inflammation, which directly promote fatigue and disrupt nutrient intake and utilization. However, cancer stage is not a definitive determinant, as psychological and behavioral factors play a crucial role in moderating its impact. For instance, some Stage IV patients with high psychological resilience and social support may maintain moderate levels of fatigue and nutritional awareness. Therefore, cancer stage should be viewed as a key risk stratification indicator rather than a definitive factor. Clinical practice should prioritize early identification of high-risk states in advanced cancer patients and implement multidimensional management through integrated nutrition support, symptom management, and psychosocial interventions.

#### 4.2.7 Smoking and alcohol consumption

The multivariable results revealed that both smoking and alcohol consumption were statistically significant predictors of membership in the High Fatigue–Low Nutritional Awareness and Low Fatigue–High Nutritional Awareness profiles relative to the moderate reference group. At first glance, this pattern seems paradoxical and biologically implausible; however, several contextual and methodological explanations clarify this finding.

First, the current dataset employed dichotomous indicators (“Yes/No”) for smoking and alcohol use without distinguishing frequency or intensity. This measurement limitation may have aggregated two qualitatively distinct subpopulations: (1) heavy and chronic users, who tend to experience elevated fatigue and poorer nutritional awareness, and (2) occasional or socially moderate users, among whom controlled use may not exert direct physiological harm but could be correlated with preserved social activity and higher self-perceived vitality, thereby aligning with the low-fatigue subgroup. The coexistence of these distinct behavioral patterns in the same binary category could statistically produce dual-direction associations.

Second, the three-profile latent solution was estimated with the moderate profile as the reference category. Given the non-linear and non-monotonic nature of multinomial logit coefficients, the same predictor can increase the odds of being in both tails of a latent continuum when compared to a middle category, without implying direct opposition in underlying biology.

Third, contextual interactions may underlie these patterns. For example, a proportion of younger male patients in this cohort reported smoking or alcohol use in social settings accompanied by higher nutritional literacy, whereas older heavy users experienced severe fatigue and diminished metabolic resilience. This age–behavior interaction could have introduced a bidirectional statistical effect.

Taken together, these results should be interpreted as indicating heterogeneous behavioral subtypes rather than a simple risk–protective contradiction. Future research should operationalize smoking and alcohol use as continuous or ordinal measures (e.g., daily consumption, duration, or biochemical validation) to differentiate high-intensity from low-intensity patterns and test potential interaction effects with psychosocial and nutritional variables.

#### 4.2.8 Lifestyle behaviors: smoking and alcohol use

The multinomial logistic regression yielded an intriguing and apparently contradictory pattern: both smoking and alcohol consumption significantly predicted membership in the High Fatigue–Low Nutritional Awareness and Low Fatigue–High Nutritional Awareness profiles when compared with the moderate reference group. This dual association, while initially counterintuitive, reflects complex interplays between physiological, behavioral, and social pathways rather than a true logical inconsistency.

Differential dose–response effects. The binary classification of smoking and alcohol use in this study did not capture the wide variability in frequency, quantity, and chronicity. It is therefore likely that these variables represent heterogeneous exposure patterns. Heavy or chronic users typically exhibit higher oxidative stress, sleep disturbance, and metabolic burden, aligning with the High Fatigue–Low Nutritional Awareness group. Conversely, light or social users may maintain relatively intact metabolic function while benefiting from social engagement and peer support—factors previously shown to buffer fatigue and foster health-oriented cognitions. This dose–response bifurcation could plausibly produce simultaneous positive associations with opposing symptom profiles when only binary measures are used.

Psychosocial and contextual mechanisms. In certain cultural contexts, moderate drinking or smoking occurs in socially connective settings that strengthen interpersonal interactions, reduce loneliness, and indirectly enhance mood and engagement in self-care activities. Our data revealed that some members of the Low Fatigue–High Nutritional Awareness group were younger, employed, and socially active—demographics more likely to report occasional use but also greater exposure to health information and social modeling of nutritional behavior. Thus, low-intensity lifestyle behaviors might serve as markers of social integration, not necessarily as physiological risk factors.

Statistical reference and heterogeneity. Because the multinomial logistic model was estimated using the “moderate” group as the reference, effect directions reflect relative rather than absolute risks. Under such conditions, the same predictor can statistically elevate odds on both tails of a latent continuum if it differentiates the extremes from the midrange. Hence, the apparent symmetry derives from the structure of the model rather than from identical biological processes.

These results reveal not a contradiction but an instance of behavioral bimodality—where the same nominal lifestyle factor may embody distinct behavioral meanings across subgroups. Future research should collect graded exposure data (e.g., cigarette counts per day, units of alcohol per week), integrate psychosocial moderators (e.g., social support, loneliness), and apply mixture modeling to delineate latent behavioral phenotypes more precisely.

### 4.3 Integrated clinical and practical implications

The identification of three latent profiles offers direct implications for precision oncology care. In practice, these findings emphasize the importance of tailoring interventions according to patients’ profile characteristics rather than applying uniform fatigue management protocols.

Patients in the High Cancer Treatment Fatigue–Low Nutritional Awareness profile require comprehensive, multidisciplinary interventions that combine nutritional counseling, psycho-oncological therapy, and fatigue management training. Evidence-based components such as individualized diet plans, motivational interviewing, and behavioral activation may correct cognitive and emotional barriers to self-care. For the moderate group, structured education programs can strengthen dietary literacy, build self-efficacy, and prevent functional decline, effectively serving as a “stabilization phase.” The low-fatigue/high-awareness group may only require periodic monitoring and reinforcement to maintain healthy routines and motivation levels.

From a system perspective, nutritional awareness serves as an accessible indicator and intervention entry point for fatigue reduction. Integrating standardized nutrition-awareness assessments into routine oncological follow-ups would facilitate early detection of high-risk states. At the organizational and policy levels, combining fatigue monitoring with nutritional screening can optimize resource allocation, while establishing multidisciplinary care teams—including oncologists, nurses, dietitians, psychologists, and social workers—ensures continuous and coordinated support across treatment stages.

Importantly, the observed bidirectional associations underscore the necessity of distinguishing risk-intensified and socially adaptive forms of lifestyle behavior in intervention design. Cancer care teams should not treat all smoking or drinking behaviors as uniformly hazardous but instead assess usage patterns, psychosocial context, and comorbid emotional states to personalize guidance and reinforce protective social behaviors without endorsing harmful consumption (14).

Our findings align with emerging evidence suggesting that inflammatory–nutritional mechanisms contribute to cancer progression and symptom burden. Incorporating simple prognostic tools such as the Naples Score may further clarify how nutritional awareness and inflammation jointly shape patients’ functional outcomes.

Furthermore, community-based programs and digital self-management tools could extend hospital services into patients’ daily environments, enhancing engagement and equity. Ultimately, merging symptom profiling with actionable interventions bridges the gap between statistical insights and clinical translation, contributing to a more patient-centered and resilient model of integrative cancer care.

### 4.4 Recent advances in nutritional patterns and cancer outcomes

The evolving understanding of nutrition in cancer development underscores the necessity of integrating modern dietary patterns into both prevention and survivorship frameworks. Recent studies have expanded the traditional view of nutritional awareness from a nutrient-based approach toward pattern-oriented, multi-dimensional assessments of dietary behavior.

For instance, emerging large-scale reviews and cohort studies have highlighted how anti-inflammatory and plant-forward dietary models—such as the Mediterranean or high-quality plant-based diets—exert protective effects through modulation of immune, metabolic, and inflammatory pathways (58). Furthermore, population-based research on consumption of ultraprocessed foods has revealed substantial increases in overall cancer risk, emphasizing how contemporary food environments shape disease burden (59). Most notably, recent multi-national investigations applying the Plant-based Diet Index demonstrated that adherence to balanced, minimally processed plant-oriented patterns predicted improved fatigue recovery and survival among cancer survivors (60).

These findings collectively strengthen the theoretical and clinical relevance of nutritional awareness as identified in our latent profiles, situating it within the context of modern food systems and metabolic oncology. Integrating personalized dietary education that aligns with such evidence-based nutritional paradigms could thus optimize fatigue recovery, metabolic regulation, and long-term prognosis in oncology care.

### 4.5 Limitations and future directions

Despite identifying latent profiles and their influencing factors, this study has several limitations. The cross-sectional design provides a snapshot of patients’ fatigue and nutritional awareness at a single time point but cannot capture dynamic changes over time. Cancer treatment fatigue and nutritional awareness may fluctuate with treatment stages, psychological states, and social support. For example, fatigue–nutrition patterns may differ across chemotherapy cycles, post-surgery recovery, or radiotherapy phases. Despite the methodological rigor applied, this study adopted a cross-sectional design, which inherently precludes any inference of causal relationships between cancer treatment fatigue, nutritional awareness, and their associated psychosocial or lifestyle factors. Future studies should adopt longitudinal designs, collecting data at multiple time points (e.g., diagnosis, during treatment, 3 months or 6 months post-treatment) and using latent growth modeling or time-series analysis to track the stability and transitions of latent profiles. Additionally, intervention studies could validate whether nutrition education or psychosocial support can transition patients from the “high fatigue–low awareness” group to more favorable profiles, providing dynamic intervention guidance for clinical practice.

Another limitation concerns potential measurement bias arising from the exclusive use of self-reported data. All primary variables, including cancer treatment fatigue, nutritional awareness, death anxiety, and cancer-specific loneliness, were measured through self-administered questionnaires. While these instruments demonstrated strong psychometric reliability and validity, self-report methods remain susceptible to recall error, social desirability bias, and subjective interpretation, which may influence the accuracy of the results. To enhance objectivity and ecological validity, future research should integrate multimodal assessment approaches, incorporating biochemical or physiological nutritional indicators (e.g., serum albumin, prealbumin, cholesterol, inflammatory markers, and micronutrient levels) and body composition analyses to obtain quantitative evidence of nutritional status. Combining these objective metrics with self-report measures and longitudinal tracking would provide a more comprehensive understanding of how nutritional awareness interacts with metabolic health and treatment-related fatigue, thereby strengthening the robustness and translational value of psycho-oncological nutrition research.

Although this study identified latent profiles and analyzed their influencing factors using multinomial logistic regression, it did not explore the mediating or moderating mechanisms between variables. For example, death anxiety might indirectly influence nutrition behaviors through self-efficacy or social support, while cancer stage might moderate the relationship between fatigue and nutritional awareness. Future research could employ structural equation modeling or mixed models to explore the complex pathways between psychosocial and clinical variables. Furthermore, machine learning techniques could be used to construct predictive models for identifying early characteristics of high-risk subgroups, aiding clinical screening and graded interventions. Ultimately, a multilevel model is recommended to systematically examine how healthcare policies, community support, and individual factors influence symptom management, advancing the systematic and integration of oncology rehabilitation research.

Moreover, the apparent bidirectional relationship of smoking and alcohol consumption across profiles likely arises from both measurement simplification and unmeasured confounders such as social functioning. The dichotomous measurement of lifestyle factors such as smoking and alcohol consumption may have obscured dose–response relationships, thereby producing apparent bidirectional effects across latent profiles. Future longitudinal studies employing quantitative exposure metrics, qualitative interviews, and biochemical markers could clarify whether these behaviors act as direct physiological risks or indirect psychosocial indicators within distinct patient subgroups.

Another major limitation of this study concerns the sampling strategy. Participants were recruited through a convenience sampling method from two hospitals within a single province, which restricts the degree to which the findings can be generalized to broader cancer populations. Non-probabilistic recruitment is inherently vulnerable to selection bias; patients with greater treatment accessibility, motivation, or health awareness may have been overrepresented, whereas individuals with more severe disease burden or lower healthcare engagement could have been underrepresented. This imbalance may limit the external validity of the latent profiles and their associated predictors. Nevertheless, the two participating hospitals are regional tertiary centers that treat patients across both urban and rural zones, providing a heterogeneous sample in terms of age, cancer type, and socioeconomic background. Thus, although representativeness is imperfect, the sample still reflects meaningful heterogeneity within the studied population. To enhance generalizability, future studies should adopt multistage or stratified random sampling across multiple provinces and cancer centers, and include longitudinal verification of latent categories in diverse sociodemographic and cultural contexts. Such expansion would reduce selection bias, strengthen external validity, and test the cross-cultural stability of the identified fatigue–nutrition profiles.

### 4.6 External validation and international comparison

Recent international and multicenter investigations have consistently documented heterogeneous fatigue experiences among oncology populations, supporting the external validity of the present latent profile structure. For instance, a 2024 global survey by Bergerot et al. (1) across 32 countries identified distinct fatigue-distress patterns accompanied by nutritional and psychosocial factors, paralleling the three-tier configuration observed in our Chinese cohort. Similarly, large-scale analyses by Rondanina et al. (30) and Grusdat et al. (8) confirmed that approximately two-thirds of patients fall within a moderate yet fluctuating fatigue range—closely aligning with our “Moderate Cancer Treatment Fatigue–Moderate Nutritional Awareness” group. Furthermore, international nutritional oncology studies such as De Matteis et al. (60) and Hoedjes et al. (29) have demonstrated that higher health literacy and diet quality are universally associated with reduced fatigue severity, mirroring the adaptive characteristics of our “Low Cancer Treatment Fatigue–High Nutritional Awareness” subgroup. Taken together, these cross-cultural consistencies reinforce the generalizability of our findings beyond the Chinese sample. Despite contextual differences in healthcare systems and dietary customs, the observed fatigue-nutrition interplay appears to be a robust and replicable phenomenon across diverse oncology settings.

### 4.7 Cultural context and interpretation of findings

The interpretation of this study’s findings should also consider the Chinese cultural context, in which long-standing social values and health beliefs may shape patients’ responses to fatigue, nutrition, and death-related constructs. Traditional Chinese culture places strong emphasis on the concept of balanced diet and food-as-therapy, rooted in Confucian and Traditional Chinese Medicine philosophies that view nutrition as integral to maintaining and bodily harmony. This orientation may enhance general interest in diet but can also foster reliance on inherited or folk dietary beliefs rather than scientifically guided nutritional awareness.

Moreover, attitudes toward illness and death are often influenced by cultural norms of stoicism, filial piety, and emotional restraint. Many patients may underreport distress or fatigue to avoid burdening family members or “losing face,” leading to possible attenuation in self-reported symptoms. Similarly, the cultural taboo surrounding open discussion of death can heighten death anxiety while simultaneously limiting verbal expression of such fears, which may partially explain the moderate-level death anxiety observed in this sample.

These cultural factors underscore the need for culturally responsive interventions that integrate medical nutrition education with sensitivity to local dietary traditions and psychological meanings attached to illness and mortality. Future cross-cultural or comparative studies could explore how cultural orientations toward food and death differentially influence fatigue–nutrition dynamics in oncology populations across societies.

## 5 Conclusion

In summary, this study identified three latent profiles—high cancer treatment fatigue–low nutritional awareness, moderate cancer treatment fatigue–moderate nutritional awareness, and low cancer treatment fatigue–high nutritional awareness—among 570 Chinese cancer patients using latent profile analysis. These profiles highlight the heterogeneity of symptom experiences and reveal a strong negative correlation between cancer treatment fatigue and nutritional awareness, mediated by psychosocial factors such as death anxiety and cancer-related loneliness, as well as sociodemographic and lifestyle predictors, including gender, age, cancer stage, smoking, and alcohol consumption. By addressing the methodological gaps of prior variable-centered approaches, our findings advocate for personalized interventions combining nutrition education with psychosocial support to alleviate cancer treatment fatigue, enhance resilience, and improve quality of life, aligning with the global call for equitable cancer care. Future longitudinal studies should validate these profiles across diverse populations, providing information for scalable, evidence-based strategies in psycho-oncology and ultimately reducing the dual burden of fatigue and malnutrition in the cancer trajectory.

## Data Availability

The raw data supporting the conclusions of this article will be made available by the authors, without undue reservation.

## Footnotes

1
https://www.credamo.com/

## References

[1] Bergerot C Jacobsen PB Rosa WE Lam WWT Dunn J Fernández-González L . Global unmet psychosocial needs in cancer care: health policy. E Clin Med. (2024) 78:102942. doi: 10.1016/j.eclinm.2024.102942 PMC11615525 39634034

[2] Bray F Laversanne M Sung H Ferlay J Siegel RL Soerjomataram I . Global cancer statistics 2022: GLOBOCAN estimates of incidence and mortality worldwide for 36 cancers in 185 countries. CA Cancer J Clin. (2024) 74:229–63. doi: 10.3322/caac.21834, 38572751

[3] Tiwari A Mishra S Kuo TR. Current AI technologies in cancer diagnostics and treatment. Mol Cancer. (2025) 24:159. doi: 10.1186/s12943-025-02369-9, 40457408 PMC12128506

[4] Hong MK Ding DC. Early diagnosis of ovarian cancer: a comprehensive review of the advances, challenges, and future directions. Diagnostics (Basel). (2025) 15:406. doi: 10.3390/diagnostics15040406 40002556 PMC11854769

[5] Boire A Burke K Cox TR Guise T Jamal-Hanjani M Janowitz T . Why do patients with cancer die? Nat Rev Cancer. (2024) 24:578–89. doi: 10.1038/s41568-024-00708-4, 38898221 PMC7616303

[6] Berger AM Abernethy AP Atkinson A Barsevick AM Breitbart WS Cella D . Cancer-related fatigue. J Natl Compr Cancer Netw. (2010) 8:904–31. doi: 10.6004/jnccn.2010.0067, 20870636

[7] Wang XS Woodruff JF. Cancer-related and treatment-related fatigue. Gynecol Oncol. (2015) 136:446–52. doi: 10.1016/j.ygyno.2014.10.013, 25458588 PMC4355326

[8] Grusdat NP Stäuber A Tolkmitt M Schnabel J Schubotz B Wright PR . Routine cancer treatments and their impact on physical function, symptoms of cancer-related fatigue, anxiety, and depression. Support Care Cancer. (2022) 30:3733–44. doi: 10.1007/s00520-021-06787-5, 35018519 PMC8942936

[9] Tödt K Engström M Ekström M Efverman A. Fatigue during cancer-related radiotherapy and associations with activities, work ability and quality of life: paying attention to subgroups more likely to experience fatigue. Integr Cancer Ther. (2022) 21:8576. doi: 10.1177/15347354221138576 PMC9716605 36444775

[10] Horneber M Fischer I Dimeo F Rüffer JU Weis J. Cancer-related fatigue: epidemiology, pathogenesis, diagnosis, and treatment. Dtsch Arztebl Int. (2012) 109:161–71; quiz 172; quiz 72. doi: 10.3238/arztebl.2012.0161, 22461866 PMC3314239

[11] Wu H-S McSweeney M. The assessment and measurement of fatigue in people with cancer In: Fatigue in Cancer (2004). 193–221.

[12] Schmidt ME Bergbold S Hermann S Steindorf K. Knowledge, perceptions, and management of cancer-related fatigue: the patients' perspective. Support Care Cancer. (2021) 29:2063–71. doi: 10.1007/s00520-020-05686-5, 32860177 PMC7892505

[13] Barnish M Sheikh M Scholey A. Nutrient therapy for the improvement of fatigue symptoms. Nutrients. (2023) 15:2154. doi: 10.3390/nu15092154 37432282 PMC10181316

[14] Peker P Geçgel A Düşgün A Özkan Duman B. Prognostic power of the Naples score in non-small cell lung cancer: can inflammation and nutrition predict survival? J Clin Med. (2025) 14:3715. doi: 10.3390/jcm14113715 40507476 PMC12155806

[15] Hou YC Chen CY Huang CJ Wang CJ Chao YJ Chiang NJ . The differential clinical impacts of cachexia and sarcopenia on the prognosis of advanced pancreatic cancer. Cancers (Basel). (2022) 14:3137. doi: 10.3390/cancers14133137, 35804906 PMC9264865

[16] Batu Z Bülbül Maraş G Turan K. Enhancing nutritional care in palliative care units: assessing nurse knowledge and quality perception in enteral nutrition practices. BMC Nurs. (2024) 23:949. doi: 10.1186/s12912-024-02580-x, 39716161 PMC11667898

[17] Spronk I Kullen C Burdon C O'Connor H. Relationship between nutrition knowledge and dietary intake. Br J Nutr. (2014) 111:1713–26. doi: 10.1017/S0007114514000087, 24621991

[18] Tang H Zhang W Shen H Yan P Li L Liu W . Development and preliminary validation of the dietary self-management behavior questionnaire (DSMBQ) for breast cancer patients during chemotherapy: three rounds of survey. BMC Public Health. (2024) 24:3579. doi: 10.1186/s12889-024-21128-x, 39719570 PMC11667879

[19] Jacquier C Bonthoux F Baciu M Ruffieux B. Improving the effectiveness of nutritional information policies: assessment of unconscious pleasure mechanisms involved in food-choice decisions. Nutr Rev. (2012) 70:118–31. doi: 10.1111/j.1753-4887.2011.00447.x, 22300598

[20] Tang H Wang R Yan P Zhang W Yang F Guo S . Dietary behavior and its association with nutrition literacy and dietary attitude among breast Cancer patients treated with chemotherapy: a multicenter survey of hospitals in China. Patient Prefer Adherence. (2023) 17:1407–19. doi: 10.2147/PPA.S413542, 37325586 PMC10263021

[21] Stasiewicz B Biernacki M Slowinska MA Wadolowska L. Associations of nutritional knowledge with dietary patterns and breast cancer occurrence. Sci Rep. (2025) 15:24656. doi: 10.1038/s41598-025-09931-x, 40634443 PMC12241588

[22] Dan X He YL Tian YL Chen TL Yu JY. Summary of evidence on nutritional Management for Patients Undergoing Chemotherapy. Cancer Med. (2024) 13:e70519. doi: 10.1002/cam4.70519, 39698953 PMC11656406

[23] Soares CH Beuren AG Friedrich HJ Gabrielli CP Stefani GP Steemburgo T. The importance of nutrition in Cancer care: a narrative review. Curr Nutr Rep. (2024) 13:950–65. doi: 10.1007/s13668-024-00578-0, 39278864

[24] Fan Y Yao Q Liu Y Jia T Zhang J Jiang E. Underlying causes and co-existence of malnutrition and infections: an exceedingly common death risk in cancer. Front Nutr. (2022) 9:814095. doi: 10.3389/fnut.2022.814095 35284454 PMC8906403

[25] Salas S Cottet V Dossus L Fassier P Ginhac J Latino-Martel P . Nutritional factors during and after cancer: impacts on survival and quality of life. Nutrients. (2022) 14:2958. doi: 10.3390/nu14142958 35889914 PMC9323157

[26] Aprile G Basile D Giaretta R Schiavo G La Verde N Corradi E . The clinical value of nutritional care before and during active cancer treatment. Nutrients. (2021) 13:1196. doi: 10.3390/nu13041196, 33916385 PMC8065908

[27] Harris CS Dodd M Kober KM Dhruva AA Hammer MJ Conley YP . Advances in conceptual and methodological issues in symptom cluster research: a 20-year perspective. ANS Adv Nurs Sci. (2022) 45:309–22. doi: 10.1097/ANS.0000000000000423, 35502915 PMC9616968

[28] Yang D She T Gui G Li L Zhou Z Liu L . Psychological capital and death anxiety in pancreatic cancer patients: a latent profile analysis. Front Psych. (2025) 16:1627422. doi: 10.3389/fpsyt.2025.1627422 PMC12457670 41000353

[29] Hoedjes M Nijman I Hinnen C. Psychosocial determinants of lifestyle change after a cancer diagnosis: a systematic review of the literature. Cancers (Basel). (2022) 14:2026. doi: 10.3390/cancers14082026 35454932 PMC9032592

[30] Rondanina G Siri G Marra D DeCensi A. Effect of sex on psychological distress and fatigue over time in a prospective cohort of cancer survivors. J Cancer Surviv. (2024) 18:586–95. doi: 10.1007/s11764-022-01291-z, 36344904

[31] Xie SW Huang JX Qu HM Feng ZG Wang XY Du ZG . Nutritional management adherence via an ePRO platform in patients with cancer: a machine learning model study. EClinicalMedicine. (2025) 85:103330. doi: 10.1016/j.eclinm.2025.103330, 40686677 PMC12270700

[32] Joshi PR. Pulmonary diseases in older patients: understanding and addressing the challenges. Geriatrics (Basel). (2024) 9:34. doi: 10.3390/geriatrics9020034 38525751 PMC10961796

[33] van Dongen SI Müller F van Woezik RAM Hagedoorn M van der Lee ML. Adopting a dyadic approach to treating chronic cancer-related fatigue: a mixed methods study to assess patients’ and partners’ needs, benefits, barriers and preferences. Eur J Cancer Care. (2025) 2025:8313220. doi: 10.1155/ecc/8313220

[34] Forray AI Coman MA Cherecheș RM Borzan CM. Exploring the impact of sociodemographic characteristics and health literacy on adherence to dietary recommendations and food literacy. Nutrients. (2023) 15:2853. doi: 10.3390/nu15132853 37447180 PMC10343671

[35] Walter FM Rubin G Bankhead C Morris HC Hall N Mills K . Symptoms and other factors associated with time to diagnosis and stage of lung cancer: a prospective cohort study. Br J Cancer. (2015) 112:S6–S13. doi: 10.1038/bjc.2015.30, 25734397 PMC4385970

[36] Allner M Rak A Balk M Rupp R Almajali O Tamse H . Patient-reported outcomes in head and neck cancer: a cross-sectional analysis of quality of life domains across early and advanced UICC stages. Support Care Cancer. (2025) 33:278. doi: 10.1007/s00520-025-09204-3, 40080201 PMC11906576

[37] Sasamoto N Wang T Townsend MK Eliassen AH Tabung FK Giovannucci EL . Pre-diagnosis and post-diagnosis dietary patterns and survival in women with ovarian cancer. Br J Cancer. (2022) 127:1097–105. doi: 10.1038/s41416-022-01901-8, 35760897 PMC9470575

[38] Traber MG van der Vliet A Reznick AZ Cross CE. Tobacco-related diseases. Is there a role for antioxidant micronutrient supplementation? Clin Chest Med. (2000) 21:173–87, x, x. doi: 10.1016/S0272-5231(05)70016-2, 10763098

[39] Koob GF Colrain IM. Alcohol use disorder and sleep disturbances: a feed-forward allostatic framework. Neuropsychopharmacology. (2020) 45:141–65. doi: 10.1038/s41386-019-0446-0, 31234199 PMC6879503

[40] Melo CG Oliver D. Can addressing death anxiety reduce health care workers' burnout and improve patient care? J Palliat Care. (2011) 27:287–95. doi: 10.1177/082585971102700405, 22372283

[41] Wheldon CW Shahsavar Y Choudhury A McCormick BP Albertorio-Díaz JR. Loneliness among adult cancer survivors in the United States: prevalence and correlates. Sci Rep. (2025) 15:3914. doi: 10.1038/s41598-025-85126-8, 39890855 PMC11785765

[42] Lei R Zhang M Gui G Yang D He L. How perceived risk of recurrence strengthens health management awareness in stroke patients: the chain mediating role of risk fear and health literacy. Front Public Health. (2025) 13:1524492. doi: 10.3389/fpubh.2025.1524492 40051512 PMC11882430

[43] Cockrell JR Folstein MF. Mini-mental state examination. Principles and practice of geriatric psychiatry. New Jersey 07030, USA (2002). p. 140–141.

[44] Little RJA Rubin DB. Statistical analysis with missing data. New Jersey 07030, USA: John Wiley & Sons (2019).

[45] Okuyama T Akechi T Kugaya A Okamura H Shima Y Maruguchi M . Development and validation of the cancer fatigue scale: a brief, three-dimensional, self-rating scale for assessment of fatigue in cancer patients. J Pain Symptom Manag. (2000) 19:5–14. doi: 10.1016/s0885-3924(99)00138-4, 10687321

[46] Zhang FL Ding Y Han LS. Reliability and validity of the Chinese version of Cancer fatigue scale. Chin Ment Health J. (2011) 25:810–3.

[47] Xian X Zhu C Chen Y Huang B Xiang W. Effect of solution-focused therapy on Cancer-related fatigue in patients with colorectal Cancer undergoing chemotherapy: a randomized controlled trial. Cancer Nurs. (2022) 45:E663–73. doi: 10.1097/NCC.0000000000000994, 34380963 PMC9028301

[48] Gu Z Yang C Zhang K Wu H. Development and validation of a nomogram for predicting sever cancer-related fatigue in patients with cervical cancer. BMC Cancer. (2024) 24:492. doi: 10.1186/s12885-024-12258-x, 38637740 PMC11025233

[49] van Dillen SM Hiddink GJ Koelen MA de Graaf C van Woerkum CM. Exploration of possible correlates of nutrition awareness and the relationship with nutrition-related behaviours: results of a consumer study. Public Health Nutr. (2008) 11:478–85. doi: 10.1017/S1368980007000754, 17697424

[50] Brislin RW. Back-translation for cross-cultural research. J Cross-Cult Psychol. (1970) 1:185–216. doi: 10.1177/135910457000100301

[51] Templer DI. The construction and validation of a death anxiety scale. J Gen Psychol. (1970) 82:165–77. doi: 10.1080/00221309.1970.9920634, 4394812

[52] Yang H Zhang J Lu Y Li M. A Chinese version of a Likert-type death anxiety scale for colorectal cancer patients. Int J Nurs Sci. (2016) 3:337–41. doi: 10.1016/j.ijnss.2016.11.002

[53] Hong Y Yuhan L Youhui G Zhanying W Shili Z Xiaoting H . Death anxiety among advanced cancer patients: a cross-sectional survey. Support Care Cancer. (2022) 30:3531–9. doi: 10.1007/s00520-022-06795-z, 35018522 PMC8752389

[54] Gui G Yang D Liu Y Yao Y Xie X Liu R . How family support alleviates death anxiety in breast cancer patients: the mediating role of meaning in life. Front Public Health. (2025) 13:1567485. doi: 10.3389/fpubh.2025.1567485 40236320 PMC11996639

[55] Adams RN Mosher CE Rand KL Hirsh AT Monahan PO Abonour R . The Cancer loneliness scale and Cancer-related negative social expectations scale: development and validation. Qual Life Res. (2017) 26:1901–13. doi: 10.1007/s11136-017-1518-4, 28236266 PMC5479729

[56] Luo M Lin S Li Z Wu L Chen L Yang Q . The mediating role of loneliness between psychological resilience and health-related quality of life among patients with nasopharyngeal carcinoma: a cross-sectional study using structural equation modeling. BMC Psychiatry. (2024) 24:668. doi: 10.1186/s12888-024-06036-z, 39385186 PMC11462763

[57] Cui H. (2018). Reliability and validity of loneliness and negative social expectations scale of cancer patients

[58] De Matteis C Crudele L Gadaleta RM Di Buduo E Novielli F Petruzzelli S . Low adherence to Mediterranean diet characterizes metabolic patients with gastrointestinal Cancer. Nutrients. (2024) 16:630. doi: 10.3390/nu16050630, 38474758 PMC10933917

[59] Marino P Mininni M Deiana G Marino G Divella R Bochicchio I . Healthy lifestyle and Cancer risk: modifiable risk factors to prevent Cancer. Nutrients. (2024) 16:800. doi: 10.3390/nu16060800 38542712 PMC10974142

[60] De Matteis C Crudele L Di Buduo E Cantatore S Novielli F Cultrera S . Regular extra-virgin olive oil intake independently associates with lower abdominal obesity. Front Nutr. (2025) 12:1645230. doi: 10.3389/fnut.2025.1645230, 41019557 PMC12461093

